# Physiological and Immunological Causes of the Susceptibility of Chronic Inflammatory Patients to COVID-19 Infection: Focus on Diabetes

**DOI:** 10.3389/fendo.2021.576412

**Published:** 2021-03-04

**Authors:** Nasim Rahmani-Kukia, Ardeshir Abbasi

**Affiliations:** ^1^ Department of Biochemistry, School of Medicine, Shiraz University of Medical Sciences, Shiraz, Iran; ^2^ Department of Immunology, Faculty of Medical Sciences, Tarbiat Modares University, Tehran, Iran

**Keywords:** COVID-19, diabetes, ACE2, chronic inflammatory disease, immune responses

## Abstract

The coronavirus disease 2019 (COVID-19) pandemic has recently emerged, which was then spread rapidly in more than 190 countries worldwide so far. According to the World Health Organization, 3,232,062 global cases of COVID-19 were confirmed on April 30^th^ with a mortality rate of 3.4%. Notably, the symptoms are almost similar to those of flu such as fever, cough, and fatigue. Unfortunately, the global rates of morbidity and mortality caused by this disease are more and still increasing on a daily basis. The rates for patients suffering from inflammatory diseases like diabetes, is even further, due to their susceptibility to the pathogenesis of COVID-19. In this review, we attempted to focus on diabetes to clarify the physiological and immunological characteristics of diabetics before and after the infection with COVID-19. We hope these conceptions could provide a better understanding of the mechanisms involved in COVID-19 susceptibility and increase the awareness of risk to motivate behavior changes in vulnerable people for enhancing the prevention. Up to now, the important role of immune responses, especially the innate ones, in the development of the worst signs in COVID-19 infection have been confirmed. Therefore, to better control patients with COVID-19, it is recommended to consider a history of chronic inflammatory diseases as well as the way of controlling immune response in these patients.

## Introduction and Epidemiology of COVID-19

In December 2019, an unknown pneumonia was identified in Wuhan, China. In past years, two pneumonia-related to coronaviruses have been appeared with the name of severe acute respiratory syndrome coronavirus (SARS-CoV) in 2002 and as Middle East respiratory syndrome coronavirus (MERS-CoV) in 2012, which infected nearly 8,422 and 1,600 people and resulted in 916 and 574 individuals’ death, respectively (1, 2). Chinese scientists identified new coronavirus on January 7^th^ as SARS-CoV-2 (3). Afterward, the World Health Organization (WHO) in February 2020 named it coronavirus disease 2019 (COVID-19) (4). The clinical spectrum of SARS-CoV-2 is similar to SARS, which includes systemic infection, respiratory tract involvement, and pneumonia associated with respiratory failure and eventually death (5). Moreover, the most important symptoms of this infection are fever, cough, and fatigue. COVID-19 has been rapidly spread in more than 200 countries worldwide and the rates of its morbidity and mortality are globally increasing on a daily basis. In this regard, WHO has reported 3,232,062 cases of COVID-19 up to April 30^th^ 2020 and confirmed 3.4% mortality rate worldwide. These mortality rates vary due to geographical location, age, and other factors in different countries (6). The comorbidity of chronic diseases such as hypertension, diabetes, and cardiovascular and respiratory diseases can increase the risk of severity and mortality in a way that these susceptibilities can be associated with the pathogenesis of COVID-19 infection. Notably, chronic diseases have several common features such as inflammatory state, immune response complications, and a higher susceptibility to infectious diseases. In a systematic study performed in February 25, 2020 on 46,248 patients, the average age to be affected by COVID-19 was shown 48 years old with the clinical symptoms of cough (86–97%), fever (59–76%), fatigue (34–68%), and shortness of breath (21–40%). Moreover, in terms of comorbidity, the prevalence rates for severe COVID-19 were as follows: hypertension (22–24%), diabetes (6–11%), cardiovascular disease (4–7%), and respiratory system diseases (1–3%), which are higher compared to non-severe patients (7). In another study conducted on 1,527 COVID-19 patients with chronic diseases, the comorbid conditions were hypertension (17.1%), cardiovascular disease (16.4%), and diabetes (9.7%) (8). In addition, on 637 MERS-CoV patients, a 50% prevalence rate for both diabetes and hypertension was verified (9). Out of 41 cases of COVID-19 in Wuhan-China, 20% of patients had diabetes comorbidity (10). Therefore, comorbidity of diabetes mellitus is one of the most common COVID-19-related death responsiveness. Since understanding the causes of high susceptibility to COVID-19 can guide the vulnerable population to evaluate the infection risk and sever outcomes, the aim of this study is to review the role of diabetes in facilitating the improvement of COVID-19.

## Diabetes and COVID-19

Diabetes as a complex metabolic and maybe an inflammatory disorder is characterized by hyperglycemia. If hyperglycemia prolongs for a long time, pro-inflammatory responses can be triggered, especially in macrophages. This can enhance the risk of diabetes-associated complications (such as cardiovascular and heart diseases), and being infected and seriously ill from infections (11). Diabetes is pathologically classified into type 1 (T1D) and type 2 (T2D). However, regardless of the type of diabetes, the severity of illness differs according to the age, complications, and how well it is controlled. It has been documented that old aged patients with pre-existing comorbidities (obesity, hypertension, heart disease, etc.), uncontrolled diabetes, and high inflammatory factors are more disposed to severe illness from COVID-19 and even display a high mortality rate from it (12). However, the interaction between diabetes and COVID-19 is reciprocal. As people with diabetes are more susceptible to COVID-19, infection with SARS-CoV-2 also can exacerbate the dysglycemia, inflammatory responses, and diabetic complications such as diabetic ketoacidosis (13) and hypokalemia, which increase the risk of being critically ill (14).

On the other hand, obesity as a general comorbidity with diabetes, improves the systemic chronic inflammation by affecting the both innate and adaptive immune systems as well as the level of IL-6 and even TNF-α (15). It has been indicated that both diabetes and obesity can prompt the cytokine storm (16) and also impair the coagulation system and thrombotic mechanisms (17), which are also identified in COVID-19 (18). Therefore, infection with SARS-CoV-2 in these patients can exacerbate the pre-existent pro-inflammatory condition, which in turn can improve the cytokine storm as well as several organ dysfunctions. Therefore, the following guidelines (**Table 1**) are recommended to be considered in patients:

**Table 1:** Regarding for Diabetics and people with chronic diseases before and during comorbidity with COVID-19.

Subject	Description Subject	Reference
**Hyperglycemia**	Hyperglycemia can increase the risk of severe outcomes resulting from COVID-19, the length of hospitalization, and even the risk of death, which can be reduced by glycemic control.	(19, 20)
**Nutrition**	Due to the same nutrition of comorbidity diabetic patients with COVID-19 and other COVID-19 patients, careful attention should be paid to nutrition, minerals, proteins, and vitamins in these patients.	(21)
**Exercise**	Lack of exercise can reduce the immune response of these patients; therefore, a proper indoor exercise is needed to regulate and strengthen the immune system in the patients affected by COVID-19.	(22)
**Vaccination**	Because of similarities in the functional mechanism of both flu and COVID-19 infections, vaccination may reduce the risk of COVID-19. This suggestion is supported by a study performed on 91,605 diabetics after the flu vaccination, and reported a reduction in the prevalence rate of pneumonia by 55% in patients over the age of 65 years old and by 43% in those under the age of 65 years old. COVID-19 also has some flu-like complications (such as fever, cough, and fatigue), which may be reduced to some extent by flu vaccination.	(23–25)
**Stress**	Stress in comorbidity diabetic patients with COVID-19 increases blood sugar level and exacerbates mortality in these patients. Therefore, anxiety and stress should be controlled and also reduced.	(26–28)
**Pancreatic tissue**	Pancreatic tissue is known as a potential target for viral in patients, leading to the impaired glucose metabolism. In COVID-19, damage to this tissue is better to be considered.	(29, 30)

## Association Between Angiotensin-Converting Enzyme 2 and COVID-19

Angiotensin-converting enzyme 2 (ACE2) is a membrane aminopeptidase presented on the surface of cardiovascular, kidney, intestinal, immune, and lung endothelial cells. ACE2 can degrade angiotensin II and produce angiotensin 1-7. Angiotensin 1-7 unlike angiotensin II has anti-oxidant, anti-fibrotic, and anti-inflammatory properties as well as vasodilatory activity. Therefore, ACE2 can protect the lung from severe respiratory injuries like acute respiratory distress syndrome (ARDS) caused by COVID-19. ACE2 is also identified as a functional receptor and target for SARS-CoV and SARS-CoV-2 (31, 32). Correspondingly, the molecular modeling has revealed some similarities between these two coronavirus receptors (33). Thus, one way of viral infection is the pneumonia caused by the binding of virus to ACE2 on the surface of lung epithelial cells (18). Throughout SARS-CoV-2 entrance, ACE2 becomes downregulated, which consequently results in a high ACE/ACE2 ratio contributing to the progression of pre-exciting pro-inflammatory responses and subsequently worsen outcomes from COVID-19 (34). Indeed, the expression of ACE2 becomes down regulated in diabetics as a consequence of the glycosylation process, which might be considered as a contributory factor for the severity of lung complications in comorbidity with SARS-CoV-2 invasion (35). Likewise, the ratio of ACE/ACE2 is found to be significantly higher in diabetics compared to healthy controls. Moreover, the relationship among ACE/ACE2 level and the increased blood pressure, fasting blood glucose, serum creatinine, and hemoglobin A1c (HbA1c) has also been demonstrated (36). As a result, it seems that there is a correlation between ACE2 level and susceptibility to COVID-19 in diabetic, which is highly debatable, so it should be considered in these patients.

## Immune Cells in Diabetic Patients

The balance among immune cells is essential for preserving homeostasis and the best performance of immune responses, however this balance can be impaired in diabetics. Several studies have revealed the increased pathological function of TCD4 cells in obesity, insulin resistance, and diabetes. TCD4 effector cells can be divided into inflammatory [T helper1 (Th1) and Th17) and anti-inflammatory (Th2 and regulatory Th (Treg)] cells with their specific cytokine secretions (37). It has been demonstrated that in adipose tissue and peripheral blood of diabetic patients, CD4 cells tend to be polarized into Th1 and Th17 inflammatory cells. However, the anti-inflammatory polarization of Th2 and its secreted cytokines (IL-4, IL-5, and IL-13) is reduced (37, 38). Moreover, in diabetes, it has been found that the Th1/Th2 ratio and the level of the related cytokines (IL-4, IL-10, IL-13, and IFN-γ) become remarkably high, whereas the anti-oxidant level decreases (39). On the other hand, it has been proved that the increased cytokine production by Th1 (IFN-γ, IL-2, TNF-α, and TNF-β) and Th17 (IL-6, IL-17A, and IL-17F) in diabetics can affect the HbA1c level (40).

Treg as a regulator of immune responses, constitutes about 5–20% of overall CD4 cells which is characterized by CD4, CD25, and Foxp3. Moreover, Treg plays a very essential role in suppressing T effector cells, inflammatory responses, and protecting against autoimmunity (41, 42). The activation of these cells leads to the secretion of IL-10, and TGF-β as well as the expression of co-inhibitory molecule cytotoxic T lymphocyte antigen-4 (CTLA-4) on their surface in order to inactivate the T effector cells. It has been shown that Treg is decreased in diabetes (43). Furthermore, the balance between Treg and Th1 or Th17 is very important in diabetic patients. Because, the balance between Treg/Th17 and Treg/Th1 ratio decrease in patients suffering from T2D (44). T CD8^+^ is also activated against infection and by releasing IFN-γ and TNF-α cytokines, which consequently increases the antiviral responses. However, an elevation in the level of T CD8^+^ has been verified in diabetes (45).

Gamma delta T (Tγδ) cells also play an important role in generating chronic inflammation by secreting cytokines (like IFN-γ and TNF-α) and affecting the function of other immune cells such as macrophages, cytotoxic T lymphocytes, Th1, Th2, Treg, and Th17 cells in the diabetics (46). It has been proved that natural killer t (NKT) cells which produce some cytokines such as IL-4 and IFN-γ are decreased in diabetic patients. Reduction of NKT cells leads to increase in the level of M1 macrophages, insulin resistance, and glucose intolerance (47). In addition, the enhancement of B cells, which play key roles in the development of insulin resistance following the production of IgG, activations of macrophages and T cells have been demonstrated in diabetes (48). NK cells, as a type of immune cells are divided into the following two subsets based on CD56 marker: dim and bright. CD56^dim^ NK cells have cytotoxic effects, while, CD56^bright^ ones are more likely to produce pro-inflammatory cytokines. In chronic inflammatory diseases like diabetes, CD56^bright^ NK cells are dominant which, release more inflammatory cytokines (49).

**Figure 1:**
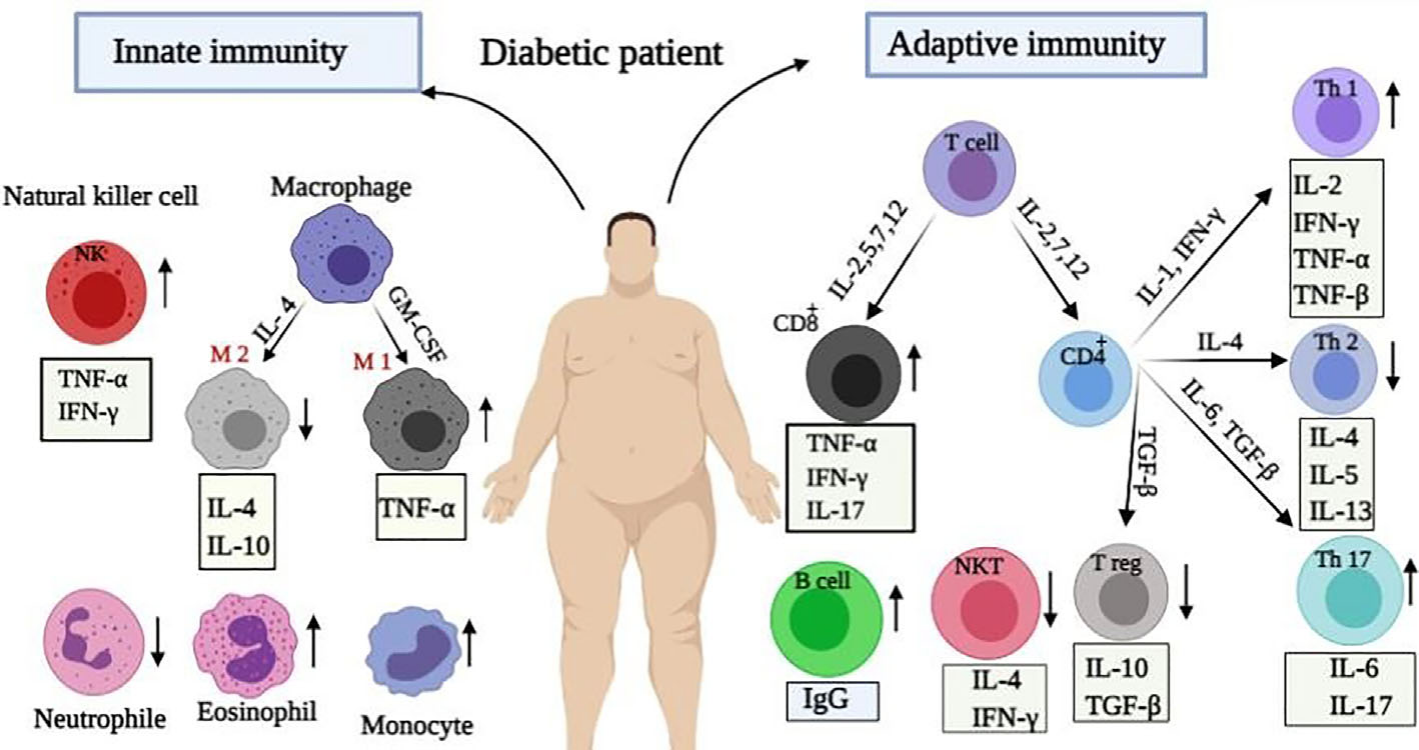
Immunological alteration in diabetic patients. Immune responses change during diabetices. Changes in innate immunity include: increase in the plasma levels of macrophages, NK cells and eosinophils, as well as their secreted cytokines with a reduction in the number of neutrophils. Moreover, adaptive immunity changes included: enhancement in Th1, Th17, TCD8^+^ cells, and released cytokines whereas reduction in Th2, Treg, and NKT cells.

Changes in the myeloid cells such as macrophages, monocytes, neutrophils, eosinophils, and basophils happen in diabetic patients as well. It has been revealed that most macrophages in the adipose tissue of diabetics are M1 macrophages, which play key functions during the pathological processes by secreting TNF-α and triggering chronic inflammation in these patients (50). Additionally, the elevation of monocytes, as effective cells on the reduction of neutrophils and eosinophils numbers as well as on the development of inflammation is perceived in diabetics (51, 52). This imbalance in the immune system of diabetics increases their susceptibility to viral pneumonia as shown in **Figure 1**. An obvious pro-inflammatory Th1 and Th17 responses was detected in the cytokine profile of SARS patients with diabetes. Similarly a significant increase has been identified in IFN-γ and IL-17A level in the lungs of patients infected with MERS-CoV and diabetes (53, 54). Therefore, it seems that any imbalance in the immune responses can increase people’s susceptibility to viral infections.

## Immune Response in COVID-19 Infection

**Figure 2:**
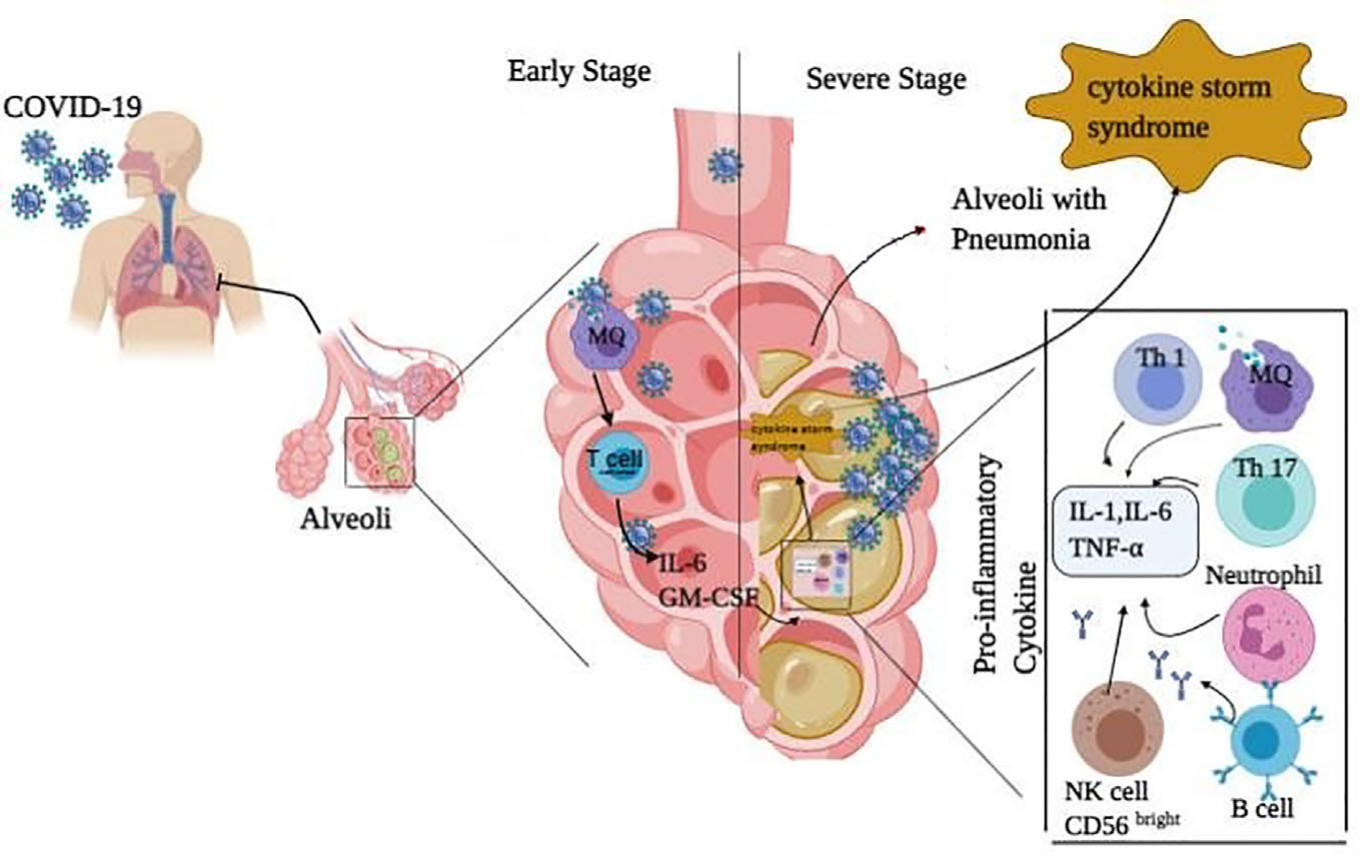
Immune response in COVID-19 infected people. The immune responses against COVID-19 infection are divided into two phases: non-severe and severe ones. In the non-sever stage response, activated T cells and macrophages release IFN-I (α, β) to remove the virus and prevent entry to severe phase. In the severe stage of infection, inflammation occurs in the lung which may increase the penetration of neutrophils, macrophages, Th1, Th17, B cells, and NK (CD56^bright^) cells with their secreted pro-inflammatory cytokines (IL-1, IL-6, and TNF-α) into the lung. This is followed by cytokine release syndrome and pneumonia in the lung.

Immune responses to COVID-19 can be divided into two phases as follows: non-severe and severe. At non-sever response, immune system tries to eliminate the virus by IFN-I (α/β) and avoids the stage’s progression to the severe phase. At severe response, inflammation arises in the lung, which may be due to impaired IFN-I regulation. Overproduction of IFN-I increases the penetration of neutrophils, macrophages, and inflammatory factors into the lung, which is then followed by cytokine storm syndrome, as well (**Figure 2**). Since chronic inflammation is generated in the body of patients with chronic diseases like diabetes, the balance of the immune system becomes dis-regulated and the inflammatory state potentially perseveres. In this situation, the numbers of Th1, Th17, M1 macrophage, NK cells (most in the affected area are probably CD56^bright^) and their secreted inflammatory cytokines increase. Moreover, the negative regulation of Treg and its secreted cytokines is decreased, which result in the loss of control and homeostasis of immune responses. All of these can heighten the diabetic patients’ susceptibility to severe COVID-19. On the other hand, SARS-CoV-2 invasion to lung enhances the influx of activated neutrophils and macrophages. An increase in the content of activated immune cells at the site of infection and their released inflammatory cytokines triggers cytokine storm. Subsequently, serious pathological complications induced in the patients’ lung can worsen the illness from COVID-19 and even terminate the life. This phenomenon is supported by some clinical studies. Accordingly, experimental findings of a study on patients with severe COVID-19 infection in China showed an elevation in neutrophils level and reduction in lymphocytes, monocytes, eosinophils, and basophils, which are followed by a sharp increase in the pro-inflammatory cytokines profile. Besides, the B, T, NK cells, and Treg depletion have been observed in patients compared to normal cases (55). Moreover, there was an increase in the plasma levels of IL-2, IL-7, IL-10, GCSF, interferon gamma-induced protein 10 (IP-10), monocyte chemoattractant protein-1 (MCP-1), macrophage inflammatory protein 1-alpha (MIP1-α), and TNFα in ICU patients (10). High concentrations of IL-1β, IFN-γ, IP-10, and MCP-1 in COVID-19 patients have also been detected (10, 56). Notably, patients who were in the ICU had higher inflammatory factors (that can be associated with cytokine storm in severe patients) compared to other patients. These clinical findings indicated that lymphocytopenia in COVID-19 patients may be due to cytokine storm (IL-1, IL-6, and TNF-α) as well as pathological effects of leukocytes and macrophages. Overall, it can be suggested that innate immune responses are extremely involved in COVID-19 infection and these points can be considered for the treatment of COVID-19 patients with co-existence of other diseases to better control the innate immune responses.

## The Effects of Some Antidiabetic Drugs on COVID-19

Considering the above-mentioned main points stating that the immune cells pattern changes to pro-inflammatory one with high level of IL-1 and TNF-α during a long-term hyperglycemia and regarding amplified immune responses to SARS-CoV-2 invasion in the lung of COVID-19 patients, this question arises that whether antidiabetic drugs with anti-inflammatory, anti-fibrotic, and anti-oxidant effects can be beneficial on prevention from infection with SARS-CoV-2 and even suffering from severe consequence of COVID-19.

**Figure 3:**
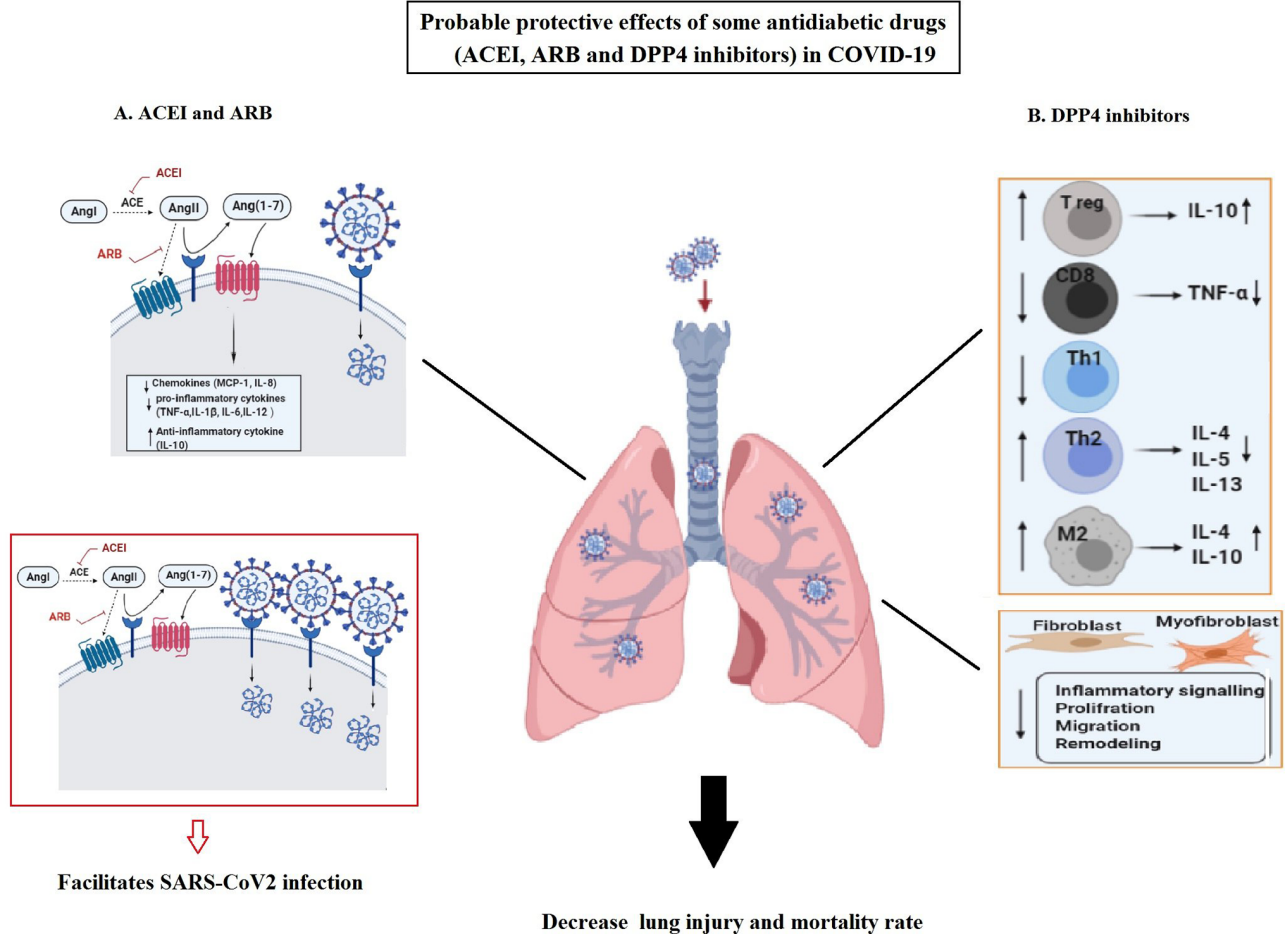
The possible protective effects of angiotensin-converting enzyme inhibitors (ACEIs), angiotensin II Type-I receptor blockers (ARBs), and also dipeptidyl peptidase 4 (DPP4) inhibitors as antidiabetic drugs in COVID-19. **(A)** Usage of ACEIs and ARB lead to over expression of ACE2. Overexpressed ACE2 can degrade angiotensin II and lead to the production of angiotensin 1-7 with favorable (anti-oxidant, anti-fibrotic, anti-inflammatory, and vasodilatory) effects in COVID-19. Its anti-inflammatory function can be related to the reduction in chemokines (MCP-1 and IL-8) and pro-inflammatory cytokines (TNF-α, IL-1β, IL-6, and IL-12) as well as increase in anti-inflammatory cytokines (IL-10) in the lung. However, the increased ACE2 can facilitate infection with SARS-CoV-2. **(B)** DPP4 inhibitors exert their anti-inflammatory impacts in the lung through regulating multiple immune cells function and anti-fibrotic effects by influencing fibroblasts and myofibroblasts activities. These effects may result in the decreased lung injury and mortality rate in COVID-19.

In this regard, several considerable theories are proposed for ACE inhibitors (ACEIs), angiotensin II Type-I receptor blockers (ARBs), and dipeptidyl peptidase 4 (DPP4) inhibitors (57). Accordingly, ACEIs and ARBs are used in patients with diabetes to prevent the risks of developing diabetes and organ failure. As a result of taking, ACE2 becomes over-expressed in these patients (58). Therefore, due to anti-inflammatory/anti-oxidant/anti-fibrotic and other promising effects of ACE2, it is suggested that the increased ACE2 may preserve them from sever outcomes of COVID-19 infection. However, there is a fear from their unfavorable impacts as well. Because, over-expressed ACE2 may also facilitate infection with SARS-CoV-2 (due to its receptor role), as displayed in **Figure 3** (13).

On the other hand, DPP4 inhibitors, as effective hyperglycemic drugs, are frequently used in diabetes. Their biological effects is not only limited to glucose metabolism, but also the anti-inflammatory and anti-fibrotic, impacts of DPP4 inhibitors on various cells have also been proved (59). DPP4 is highly expressed on lung mesothelium and various lung cells (such as alveolar cells, resident macrophages) as well as multiple immune cell types (such as macrophages, T cells, B/NK, and dendritic cells) (60). Thus, it is logical to assume DPP4 effect on their functions (57). In addition, DPP4 possesses a pro-inflammatory function by stimulating monocytes to produce IL-6 and TNF-α. Therefore, DPP4 inhibitors can also be verified as anti-inflammatory agents (60). Accordingly, their anti-inflammatory and protective impacts have been confirmed in mice and human lung injuries (61). Moreover, it is demonstrated that DPP4 inhibitors display their positive anti-inflammatory effects by reducing the high level of pro-inflammatory factors such as TNF-α, as well as elevating anti-inflammatory IL-10 in T2D patients (**Figure 3**) (62). Therefore, DPP4 inhibitors exert their anti-inflammatory functions on different cell types such as macrophages, T cells, and lung cells. In addition, it seems that DPP4 inhibitors have some anti-fibrotic effects on the lung by affecting the proliferative and inflammatory-signaling-pathways of human pulmonary arterial smooth muscle cells and fibroblasts (63).

As a result, considering the promising inflammatory and immunological impacts of DPP4 inhibitors, they might be beneficial on modulating immune and inflammatory responses in the respiratory system of COVID-19, which are abnormally amplified to minimize the severity of illness and its mortality rate. On the other hand, it is suggested that DDP4 4 inhibitors could reduce the SARS-CoV-2 internalization/replication within the airway due to the estimated co-receptor role of DPP4 (64). In this regard, more clinical data are required to confirm these findings.

## Conclusion

In chronic inflammatory diseases like diabetes, the proliferation, differentiation, distribution, and function of both the innate and adaptive immunities become dysregulated and the immune homeostatic balance becomes disrupted. Accordingly, these in turn make patients more susceptible to be infected with SARS-CoV-2, severe outcomes, and the increased mortality rate resulted from it compared to people without it. Since the activation of innate and adaptive immune responses plays an essential role in severity of symptoms and the risk of mortality from COVID-19, all efforts attempt to reduce the incidence and death rates. In this regard, we tried to focus on diabetes to clarify the physiological and immunological characteristics of diabetic patients once before and once after infecting with COVID-19. We hope it could provide better understanding on inflammatory mechanisms to better control the immune responses and its consequences in COVID-19.

## Author Contributions

AA: the original draft preparation, writing, editing, and graphic design. NR-K: writing, editing, and relevant idea for graphic design. All authors contributed to the article and approved the submitted version.

## Conflict of Interest

The authors declare that the research was conducted in the absence of any commercial or financial relationships that could be construed as a potential conflict of interest.

## References

[1] Badawi A Ryoo SG. Prevalence of diabetes in the 2009 influenza A (H1N1) and the Middle East respiratory syndrome coronavirus: a systematic review and meta-analysis. J Public Health Res (2016) 5(3):130–8. 10.4081/jphr.2016.733 PMC5206772 28083520

[2] Chan K Zheng J Mok Y Li Y LIU YN Chu C. SARS: prognosis, outcome and sequelae. Respirology (2003) 8:S36–40. 10.1046/j.1440-1843.2003.00522.x PMC7169213 15018132

[3] Phelan A Katz R Gostin L. The novel coronavirus originating in Wuhan. Challenges Global Health Governance JAMA (2020) 323(8):709–10. 10.1001/jama.2020.1097 31999307

[4] Gorbalenya AE. Severe acute respiratory syndrome-related coronavirus–The species and its viruses, a statement of the Coronavirus Study Group. BioRxiv (2020) 937862:1–16. 10.1101/2020.02.07.937862v1

[5] Bai Y Yao L Wei T Tian F Jin D-Y Chen L. Presumed asymptomatic carrier transmission of COVID-19. Jama (2020) 323(14):1406–07. 10.1001/jama.2020.2565 PMC7042844 32083643

[6] Guan W-j Ni Z-y Hu Y Liang W-h Ou C-q He J-x. Clinical characteristics of coronavirus disease 2019 in China. New Engl J Med (2020) 382:1708–20. 10.1101/2020.02.06.20020974 PMC7092819 32109013

[7] Yang J Zheng Y Gou X Pu K Chen Z Guo Q. Prevalence of comorbidities in the novel Wuhan coronavirus (COVID-19) infection: a systematic review and meta-analysis. Int J Infect Dis (2020) 30136-3:1–14. 10.1016/j.ijid.2020.03.017 PMC7194638 32173574

[8] Li B Yang J Zhao F Zhi L Wang X Liu L. Prevalence and impact of cardiovascular metabolic diseases on COVID-19 in China. Clin Res Cardiol (2020) 109:1–8. 10.1007/s00392-020-01626-9 PMC7087935 32161990

[9] Badawi A Ryoo SG. Prevalence of comorbidities in the Middle East respiratory syndrome coronavirus (MERS-CoV): a systematic review and meta-analysis. Int J Infect Dis (2016) 49:129–33. 10.1016/j.ijid.2016.06.015 PMC7110556 27352628

[10] Huang C Wang Y Li X Ren L Zhao J Hu Y. Clinical features of patients infected with 2019 novel coronavirus in Wuhan, China. Lancet (2020) 395(10223):497–506. 10.1016/S0140-6736(20)30183-5 31986264 PMC7159299

[11] Peric S Stulnig TM. Diabetes and COVID-19: Disease—Management—People. Wiener Klinische Wochenschrift (2020) 1:356–61. 10.1007/s00508-020-01672-3 PMC7238399 32435867

[12] Guo W Li M Dong Y Zhou H Zhang Z Tian C. Diabetes is a risk factor for the progression and prognosis of COVID-19. Diabetes/Metabol Res Rev (2020) 36(7):e3319. 10.1002/dmrr.3319 PMC7228407 32233013

[13] Pal R Bhadada SK. COVID-19 and diabetes mellitus: An unholy interaction of two pandemics. Diabetes Metab Syndrome: Clin Res Rev (2020) 14:513–7. 10.1016/j.dsx.2020.04.049 PMC7202837 32388331

[14] Li X Hu C Su F Dai J. Hypokalemia and clinical implications in patients with coronavirus disease 2019 (COVID-19). MedRxiv (2020) 20028530:1–22. 10.1101/2020.02.27.20028530

[15] Coelho M Oliveira T Fernandes R. Biochemistry of adipose tissue: an endocrine organ. Arch Med Sci: AMS (2013) 9(2):191. 10.5114/aoms.2013.33181 23671428 PMC3648822

[16] Papadokostaki E Tentolouris N Liberopoulos E. COVID-19 and diabetes: What does the clinician need to know? Primary Care Diabetes (2020) 14:558–63. 10.1016/j.pcd.2020.06.010 PMC7332931 32654982

[17] Kaye SM Pietiläinen KH Kotronen A Joutsi-Korhonen L Kaprio J Yki-Järvinen H. Obesity-related derangements of coagulation and fibrinolysis: a study of obesity-discordant monozygotic twin pairs. Obesity (2012) 20(1):88–94. 10.1038/oby.2011.287 21959347

[18] Magro C Mulvey JJ Berlin D Nuovo G Salvatore S Harp J. Complement associated microvascular injury and thrombosis in the pathogenesis of severe COVID-19 infection: a report of five cases. Trans Res (2020) 220:1–13. 10.1016/j.trsl.2020.04.007 PMC7158248 32299776

[19] Sardu C D’Onofrio N Balestrieri ML Barbieri M Rizzo MR Messina V. Outcomes in Patients With Hyperglycemia Affected by Covid-19: Can We Do More on Glycemic Control? Diabetes Care (2020) 43:1408–15. 10.2337/figshare.12275516.v1 PMC7305003 32430456

[20] Zhou J Tan J. Diabetes patients with COVID-19 need better blood glucose management in Wuhan, China. Metabolism (2020) 107:154216. 10.1016/j.metabol.2020.154216 32220612 PMC7102634

[21] Li T Zhang Y Gong C Wang J Liu B Shi L. Prevalence of malnutrition and analysis of related factors in elderly patients with COVID-19 in Wuhan, China. Eur J Clin Nutr (2020) 74:1–5. 10.1038/s41430-020-0642-3 PMC7175450 32322046

[22] Banerjee M Chakraborty S Pal R. Diabetes self-management amid COVID-19 pandemic. Diabetes Metab Syndrome: Clin Res Rev (2020) 14:351–4. 10.1016/j.dsx.2020.04.013 PMC7194953 32311652

[23] Patel A Jernigan DB. Initial public health response and interim clinical guidance for the 2019 novel coronavirus outbreak—United States, December 31, 2019–February 4, 2020. Morbid Mortal Weekly Rep (2020) 69(5):140. 10.15585/mmwr.mm6908e1 PMC7004396 32027631

[24] Lau D Eurich DT Majumdar SR Katz A Johnson JA. Effectiveness of influenza vaccination in working-age adults with diabetes: a population-based cohort study. Thorax (2013) 68(7):658–63. 10.1136/thoraxjnl-2012-203109 PMC3711373 23535212

[25] Goeijenbier M Van Sloten TT Slobbe L Mathieu C Van Genderen P Beyer WE. Benefits of flu vaccination for persons with diabetes mellitus: a review. Vaccine (2017) 35(38):5095–101. 10.1016/j.vaccine.2017.07.095 28807608

[26] Qiu J Shen B Zhao M Wang Z Xie B Xu Y. A nationwide survey of psychological distress among Chinese people in the COVID-19 epidemic: implications and policy recommendations. Gen Psychiatry (2020) 33(2):1–3. 10.1136/gpsych-2020-100213 PMC7061893 32215365

[27] Hartmann-Boyce J Morris E Goyder C Kinton J Perring J Nunan D. Diabetes and COVID-19: risks, management, and learnings from other national disasters. Diabetes Care (2020) 43(8):1695–703. 10.2337/dc20-1192 32546593

[28] Miazgowski T Bikowska M Ogonowski J Taszarek A. The impact of health locus of control and anxiety on self-monitored blood glucose concentration in women with gestational diabetes mellitus. J Women’s Health (2018) 27(2):209–15. 10.1089/jwh.2017.6366 28829663

[29] Cure E Cure MC. COVID−19 may affect the endocrine pancreas by activating Na+/H+ exchanger 2 and increasing lactate levels. J Endocrinol Invest (2020) 43:1167—8. 10.1007/s40618-020-01307-4 PMC8824748 32468512

[30] Wang F Wang H Fan J Zhang Y Wang H Zhao Q. Pancreatic injury patterns in patients with COVID-19 pneumonia. Gastroenterology (2020) 159:367–70. 10.1053/j.gastro.2020.03.055 PMC7118654 32247022

[31] Li F Li W Farzan M Harrison SC. Structure of SARS coronavirus spike receptor-binding domain complexed with receptor. Science (2005) 309(5742):1864–8. 10.1126/science.1116480 16166518

[32] Turner AJ Hiscox JA Hooper NM. ACE2: from vasopeptidase to SARS virus receptor. Trends Pharmacol Sci (2004) 25(6):291–4. 10.1016/j.tips.2004.04.001 PMC7119032 15165741

[33] Lu R Zhao X Li J Niu P Yang B Wu H. Genomic characterisation and epidemiology of 2019 novel coronavirus: implications for virus origins and receptor binding. Lancet (2020) 395(10224):565–74. 10.1016/S0140-6736(20)30251-8 PMC7159086 32007145

[34] Pagliaro P Penna C. ACE/ACE2 Ratio: A Key Also in 2019 Coronavirus Disease (Covid-19)? Front Med (2020) 7:1–5. 10.3389/fmed.2020.00335 PMC7314898 32626721

[35] Pal R Bhansali A. COVID-19, diabetes mellitus and ACE2: the conundrum. Diabetes Res Clin Practice (2020) 162:1–2. 10.1016/j.diabres.2020.108132 PMC7118535 32234504

[36] Mizuiri S Hemmi H Arita M Ohashi Y Tanaka Y Miyagi M. Expression of ACE and ACE2 in individuals with diabetic kidney disease and healthy controls. Am J Kidney Dis (2008) 51(4):613–23. 10.1053/j.ajkd.2007.11.022 18371537

[37] Raphael I Nalawade S Eagar TN Forsthuber TG. T cell subsets and their signature cytokines in autoimmune and inflammatory diseases. Cytokine (2015) 74(1):5–17. 10.1016/j.cyto.2014.09.011 25458968 PMC4416069

[38] Cortez-Espinosa N Cortés-Garcia JD Martínez-Leija E Rodríguez-Rivera JG Barajas-López C González-Amaro R. CD39 expression on Treg and Th17 cells is associated with metabolic factors in patients with type 2 diabetes. Hum Immunol (2015) 76(9):622–30. 10.1016/j.humimm.2015.09.007 26386144

[39] Sireesh D Dhamodharan U Ezhilarasi K Vijay V Ramkumar KM. Association of NF-E2 related factor 2 (Nrf2) and inflammatory cytokines in recent onset type 2 diabetes mellitus. Sci Rep (2018) 8(1):1–10. 10.1038/s41598-018-22913-6 29572460 PMC5865120

[40] Ip B Cilfone NA Belkina AC DeFuria J Jagannathan-Bogdan M Zhu M. Th17 cytokines differentiate obesity from obesity-associated type 2 diabetes and promote TNF α production. Obesity (2016) 24(1):102–12. 10.1002/oby.21243 PMC4688084 26576827

[41] Dimeloe S Burgener AV Grählert J. Hess C. T-cell metabolism governing activation, proliferation and differentiation; a modular view. Immunology (2017) 150(1):35–44. 10.1111/imm.12655 27479920 PMC5341500

[42] Zheng Y Rudensky AY. Foxp3 in control of the regulatory T cell lineage. Nat Immunol (2007) 8(5):457–62. 10.1038/ni1455 17440451

[43] Du X Liu M Su J Zhang P Tang F Ye P. Uncoupling therapeutic from immunotherapy-related adverse effects for safer and effective anti-CTLA-4 antibodies in CTLA4 humanized mice. Cell Res (2018) 28(4):433–47. 10.1038/s41422-018-0012-z PMC5939041 29463898

[44] Zeng L Lu H Deng H Mu P Li X Wang M. Noninferiority effects on glycemic control and β-cell function improvement in newly diagnosed type 2 diabetes patients: basal insulin monotherapy versus continuous subcutaneous insulin infusion treatment. Diabetes Technol Ther (2012) 14(1):35–42. 10.1089/dia.2011.0123 21877913 PMC3249622

[45] Monteiro-Sepulveda M Touch S Mendes-Sá C André S Poitou C Allatif O. Jejunal T cell inflammation in human obesity correlates with decreased enterocyte insulin signaling. Cell Metab (2015) 22(1):113–24. 10.1016/j.cmet.2015.05.020 26094890

[46] Rhodes KA Andrew EM Newton DJ Tramonti D. Carding SR. A subset of IL-10-producing γδ T cells protect the liver from Listeria-elicited, CD8+ T cell-mediated injury. Eur J Immunol (2008) 38(8):2274–83. 10.1002/eji.200838354 18624301

[47] Guzik TJ Skiba DS Touyz RM Harrison DG. The role of infiltrating immune cells in dysfunctional adipose tissue. Cardiovasc Res (2017) 113(9):1009–23. 10.1093/cvr/cvx108 PMC5852626 28838042

[48] Simar D Versteyhe S Donkin I Liu J Hesson L Nylander V. DNA methylation is altered in B and NK lymphocytes in obese and type 2 diabetic human. Metabolism (2014) 63(9):1188–97. 10.1016/j.metabol.2014.05.014 24996265

[49] Carvelli J Piperoglou C Bourenne J Farnarier C Banzet N Demerlé C. Imbalance of circulating innate lymphoid cell subpopulations in patients with septic shock. Front Immunol (2019) 10:2179. 10.3389/fimmu.2019.02179 31616411 PMC6763762

[50] Kim Y Bayona PW Kim M Chang J Hong S Park Y. Macrophage lamin A/C regulates inflammation and the development of obesity-induced insulin resistance. Front Immunol (2018) 9:696. 10.3389/fimmu.2018.00696 29731750 PMC5920030

[51] Kawarabayashi R Motoyama K Nakamura M Yamazaki Y Morioka T Mori K. The association between monocyte surface CD163 and insulin resistance in patients with type 2 diabetes. J Diabetes Res (2017) 2017:1–9. 10.1155/2017/6549242 PMC5763167 29445750

[52] Wu D Molofsky AB Liang H-E Ricardo-Gonzalez RR Jouihan HA Bando JK. Eosinophils sustain adipose alternatively activated macrophages associated with glucose homeostasis. Science (2011) 332(6026):243–7. 10.1126/science.1201475 PMC3144160 21436399

[53] Mahallawi WH Khabour OF Zhang Q Makhdoum HM Suliman BA. MERS-CoV infection in humans is associated with a pro-inflammatory Th1 and Th17 cytokine profile. Cytokine (2018) 104:8–13. 10.1016/j.cyto.2018.01.025 29414327 PMC7129230

[54] Kulcsar KA Coleman CM Beck SE Frieman MB. Comorbid diabetes results in immune dysregulation and enhanced disease severity following MERS-CoV infection. JCI Insight (2019) 4(20):1–18. 10.1172/jci.insight.131774 PMC6824443 31550243

[55] Qin C Zhou L Hu Z Zhang S Yang S Tao Y. Dysregulation of immune response in patients with COVID-19 in Wuhan, China. China (February 17 2020) (2020) 15:762–8. 10.1093/cid/ciaa248 PMC7108125 32161940

[56] Liu Y Zhang C Huang F Yang Y Wang F Yuan J. Elevated plasma level of selective cytokines in COVID-19 patients reflect viral load and lung injury. Natl Sci Rev (2020) 109:1–8. 10.1093/nsr/nwaa037 PMC7107806 34676126

[57] Pantanetti P Cangelosi G Ambrosio G. Potential role of incretins in diabetes and COVID-19 infection: a hypothesis worth exploring. Internal Emergency Med (2020) 15:1–4. 10.1007/s11739-020-02389-x PMC7317260 32592113

[58] Verma A Shan Z Lei B Yuan L Liu X Nakagawa T. ACE2 and Ang-(1-7) confer protection against development of diabetic retinopathy. Mol Ther (2012) 20(1):28–36. 10.1038/mt.2011.155 21792177 PMC3255596

[59] Seferović PM Coats AJ Ponikowski P Filippatos G Huelsmann M Jhund PS. European Society of Cardiology/Heart Failure Association position paper on the role and safety of new glucose-lowering drugs in patients with heart failure. Eur J Heart Failure (2020) 22(2):196–213. 10.1002/ejhf.1673 31816162

[60] Shao S Xu Q Yu X Pan R Chen Y. Dipeptidyl peptidase 4 inhibitors and their potential immune modulatory functions. Pharmacol Ther (2020) 209:107503. 10.1016/j.pharmthera.2020.107503 32061923 PMC7102585

[61] Kawasaki T Chen W Htwe YM Tatsumi K Dudek SM. DPP4 inhibition by sitagliptin attenuates LPS-induced lung injury in mice. Am J Physiol-Lung Cell Mol Physiol (2018) 315(5):L834–L45. 10.1152/ajplung.00031.2018 30188745

[62] Satoh-Asahara N Sasaki Y Wada H Tochiya M Iguchi A Nakagawachi R. A dipeptidyl peptidase-4 inhibitor, sitagliptin, exerts anti-inflammatory effects in type 2 diabetic patients. Metabolism (2013) 62(3):347–51. 10.1016/j.metabol.2012.09.004 23062489

[63] Smelcerovic A Kocic G Gajic M Tomovic K Djordjevic V Stankovic-Djordjevic D. DPP-4 Inhibitors in the Prevention/Treatment of Pulmonary Fibrosis, Heart and Kidney Injury Caused by COVID-19—A Therapeutic Approach of Choice in Type 2 Diabetic Patients? Front Pharmacol (2020) 11:1185. 10.3389/fphar.2020.01185 32848788 PMC7419672

[64] Solerte SB Di Sabatino A Galli M Fiorina P. Dipeptidyl peptidase-4 (DPP4) inhibition in COVID-19. Acta Diabetol (2020) 57(7):779–83. 10.1007/s00592-020-01539-z PMC7275134 32506195

